# Histone Deacetylase (HDAC) Inhibitors Down-Regulate Endothelial Lineage Commitment of Umbilical Cord Blood Derived Endothelial Progenitor Cells

**DOI:** 10.3390/ijms131115074

**Published:** 2012-11-15

**Authors:** Florin Iordache, Cosmin Buzila, Andrei Constantinescu, Eugen Andrei, Horia Maniu

**Affiliations:** Institute of Cellular Biology and Pathology of Romanian Academy, “Nicolae Simionescu”, 8, B.P. Hasdeu, Bucharest 050568, Romania; E-Mails: floriniordache84@yahoo.com (F.I.); cosmin.buzila@icbp.ro (C.B.); andrei.constantinescu@icbp.ro (A.C.); eugen.andrei@yahoo.com (E.A.)

**Keywords:** endothelial progenitor cells, differentiation, acetylation, histone deacetylases

## Abstract

To test the involvement of histone deacetylases (HDACs) activity in endothelial lineage progression, we investigated the effects of HDAC inhibitors on endothelial progenitors cells (EPCs) derived from umbilical cord blood (UCB). Adherent EPCs, that expressed the endothelial marker proteins (PCAM-1, CD105, CD133, and VEGFR_2_) revealed by flow cytometry were treated with three HDAC inhibitors: Butyrate (BuA), Trichostatin A (TSA), and Valproic acid (VPA). RT-PCR assay showed that HDAC inhibitors down-regulated the expression of endothelial genes such as *VE-cadherin*, *CD133*, *CXCR4* and *Tie-2*. Furthermore, flow cytometry analysis illustrated that HDAC inhibitors selectively reduce the expression of VEGFR_2_, CD117, VE-cadherin, and ICAM-1, whereas the expression of CD34 and CD45 remained unchanged, demonstrating that HDAC is involved in endothelial differentiation of progenitor cells. Real-Time PCR demonstrated that TSA down-regulated telomerase activity probably via suppression of hTERT expression, suggesting that HDAC inhibitor decreased cell proliferation. Cell motility was also decreased after treatment with HDAC inhibitors as shown by wound-healing assay. The balance of acethylation/deacethylation kept in control by the activity of HAT (histone acetyltransferases)/HDAC enzymes play an important role in differentiation of stem cells by regulating proliferation and endothelial lineage commitment.

## 1. Introduction

Epigenetic changes in the genome include DNA methylation and histone modifications (acetylation, methylation, phosphorylation, ubiquitination, sumoylation), two mechanisms that are often tightly linked in the regulation of gene expression and involved in many cellular processes [1–3]. Differentiation of endothelial progenitor cells (EPCs) is a process controlled by histone modifications that permit the fine tuning of cell fate specific gene expression [4,5]. Regarding the epigenetic changes on pluripotent stem cells, HDACs play important roles in stem cell self-renewal status, commitment and differentiation assessment. HDACs can reverse epigenetic traits that characterize genes involved in the regulation of self-renewal or differentiation and improve embryo developmental potential [1]. HDAC inhibitors down-regulated Nanog expression in undifferentiated embryonic stem cells (ESCs) and in ESCs induced to differentiate by retinoic acid for 1 or 2 days, but HDAC inhibitors had no effect on Nanog expression when ESCs were induced to differentiate by retinoic acid for 3 days. This could be due to the fact that the Nanog expression level in fully differentiated ESCs is too low to be further down-regulated. These results suggest that HDAC inhibitors may promote reprogramming partially through activating pluripotency genes at some intermediate stages [4,6].

The balance of acetylation/deacetylation kept in control by the activity of HAT (histone acetyltransferases) and HDAC (histone deacetylases) enzymes and play an important role in differentiation of endothelial progenitors by regulating proliferation and endothelial lineage progression [5–7]. Histone deacetylases (HDACs) are cofactors for the regulation of gene transcription. Human HDACs are grouped into four classes: Class I HDAC (1, 2, 3 and 8), with the possible exception of HDAC3, are predominantly nuclear in localization. Class II HDACs (HDACs 4, 5, 6, 7, 9 and 10) have a high degree of homology to the yeast HDAC *Hda-1*, are larger in size (120–150 kDa) compared to Class I HDACs, and are expressed in both the nucleus and cytoplasm. HDAC11, which shares some but not sufficient homology to both Class I and II HDACs is assigned to its own class, Class IV. All these classes have zinc containing catalytic domain, compared with Class III (sirtuins) that needs NAD as cofactor for fulfill its function [8,9]. Nonselective inhibitors of Class I and Class II HDACs reduce tube formation of endothelial cells *in vitro*, inhibit postnatal neovascularization in response to hypoxia, and block tumor angiogenesis [10].

Despite advances in uncovering the molecular basis of these epigenetic mechanisms, their role in cardiovascular development, homeostasis and disease, and stem cell biology remain unclear [4]. The isolation and characterization of endothelial progenitor cells from peripheral blood was first reported by Asahara *et al.* in 1997. As the cells differentiate, they acquire endothelial lineage markers, such as vascular endothelium-cadherin, PECAM-1 (CD31), von Willebrand factor, endothelial nitric oxide synthase (eNOS), E-selectin, and vascular endothelial growth factor receptor (VEGFR2), and incorporate acetylated low-density lipoprotein cholesterol [11,12]. Because mature endothelial cells have limited ability to regenerate damaged endothelium since these cells are terminally differentiated, EPCs may play a significant role in vascular repair and healing, and reduce cardiovascular events associated with endothelial cell loss, including thrombosis, restenosis, and hypertension [13–15]. Understanding the mechanism induced by histones modifications that regulates pluripotency and differentiation of EPCs may help stem cell research for the elaboration of new hopeful treatments for various cardiovascular, neurodegenerative, autoimmune diseases, spinal cord injuries, by transplantation therapies of progenitor’s cells [16–18].

The aim of this study was to investigate the role of acetylation in endothelial lineage progression using HDAC inhibitors and to show that this process is necessary for endothelial commitment of progenitor cells.

## 2. Results and Discussion

### 2.1. EPC Proliferation and Motility

The telomeres of human cells are composed of tandem repeats of the sequence 5′-GGTTAG-3′ and protect chromosomal ends from fusion events, also playing an important structural and functional role. Telomere maintenance mechanism is provided by a specialized enzyme called telomerase. In human cells, telomerase functions as a reverse transcriptase to add multiple copies of the 5′-GGTTAG-3′ motif to the end of the G-strand of the telomere. In human somatic cells, telomere length decreases with each cell division event. In the majority of tumor cells (85%–90%), this enzyme is overexpressed, correlated with a high proliferative profile [19]. Even in normal stem cells, the level of telomerase activity is lower than in cancer cells [14].

**Figure 1:**
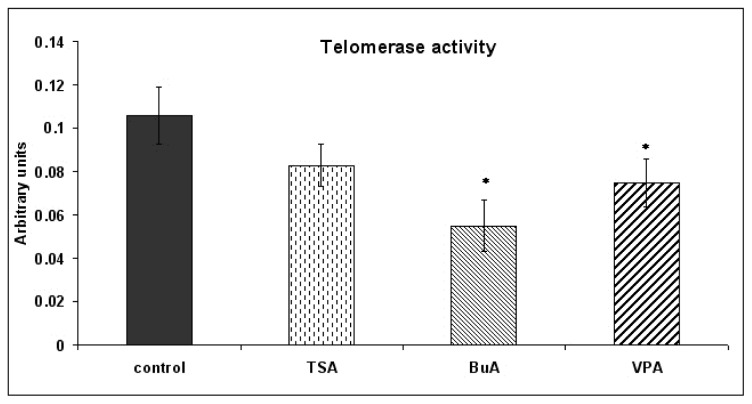
Telomerase activity in the presence of histone deacetylases (HDAC) inhibitors. All three inhibitors: Butyrate (BuA) (2 mM), Valproic acid (VPA) (0.5 mM) and Trichostatin A (TSA) (1 μM) decreased endothelial progenitors cells (EPCs) proliferation, significantly by BuA and VPA. Results are represented as mean ± standard error, *n* = 3, * *p* < 0.05.

**Figure 2:**
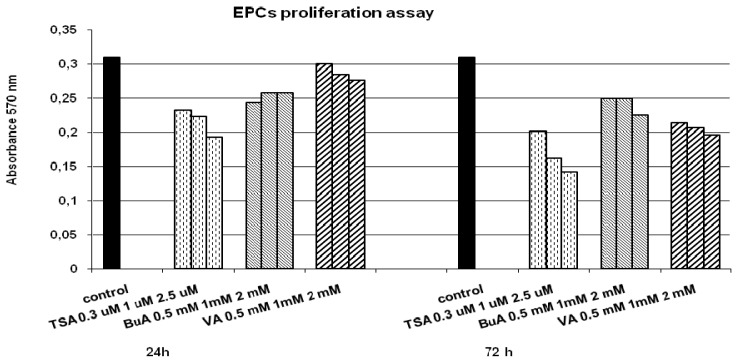
EPCs proliferation in the presence of different concentrations of HDAC inhibitors and at different time intervals.

**Figure 3:**
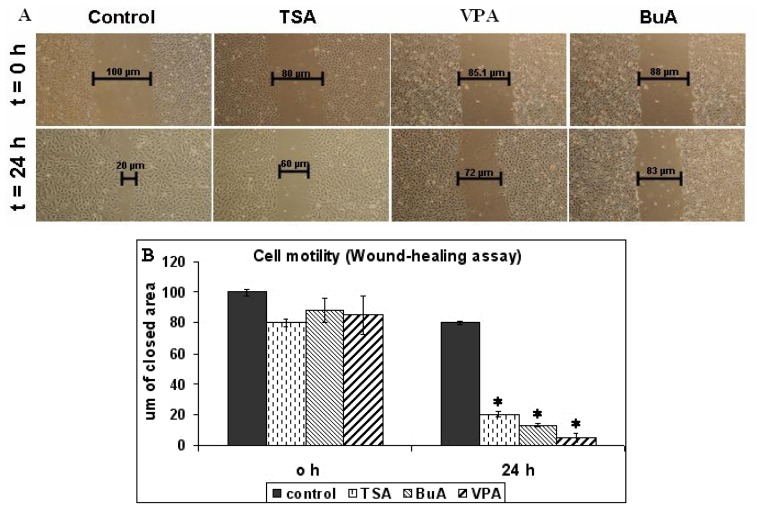
Study of EPCs migration by wound healing assay, following stimulation with HDAC inhibitors. Cell migration was quantified at 24 h after stimulation, by measuring the wounded area (in pixels) that was covered by the cells during the indicated time points (**A**, Nikon, 4×). Results are represented as mean ± standard error, *n* = 6, * *p* < 0.05, (**B**).

For evaluation of proliferative potential, we measured telomerase activity of EPCs. After being treated with HDAC inhibitors, EPCs displayed significantly diminished levels of telomerase activity compared with the control, illustrating a decreasing proliferative potential (Figure 1). Furthermore we observed that all HDAC inhibitors modulate proliferation in a dose-dependent manner (Figure 2). Cell motility, evaluated by classical wound-healing assay, at 24 h revealed that all three HDAC inhibitors decreased the migration ability of the EPCs. At 24 h after plate scratching, EPCs migration ability compared to control was reduce at a rate of 75% for TSA, 83% for BuA, and 89% for VPA respectively (Figure 3). The effect of telomerase activity on regenerative properties of endothelial progenitor cells (EPCs) in neovascularization is featured by angiogenic properties, mitogenic activity, migratory activity, and cell survival. HDAC inhibitors down-regulated telomerase activity probably via suppression of hTERT expression, leading to reduced proliferative and migratory activity of EPCs [20,21].

### 2.2. Expression of Molecules Involved in EPCs Differentiation

Endothelial progenitor cells were isolated and characterized from Umbilical Cord Blood (UCB), Wharton’s Jelly, and adult peripheral blood [11,12,22], and showed that it may express cell surface markers shared by hematopoietic stem cells (HSC) since endothelial and blood cells share a similar mesodermal origin during embryonic development. [12,22] Using monoclonal antibodies and fluorescence activated cell sorting (FACS) to searching for cells expressing CD34 and KDR, Yoder *et al.* 2009, included CD133 expression as a discriminating marker. Cells expressing CD34, KDR, and CD133 were identified from mobilized adult peripheral blood, umbilical cord blood, and human fetal liver samples. Duda *et al.* 2007, reported that EPCs can be defined as a discrete population of cells expressing CD31^+^CD45^−^CD34^dim^CD133^+^, along differentiation gaining endothelial cell markers like CD 144, VEGFR2, and Tie-2 [23].

**Figure 4:**
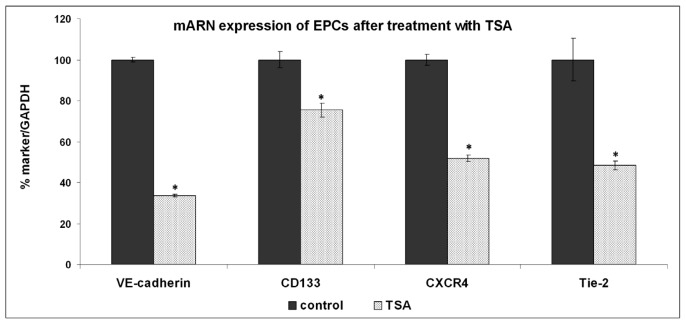
Effect of TSA (1 μM) on mRNA expression of EPCs markers. The expression of *VE-cadherin*, *CD133*, *CXCR4*, and *Tie-2* mRNA was significantly decreased. Results are represented as mean ± standard error, *n* = 3, * *p* < 0.05.

**Figure 5:**
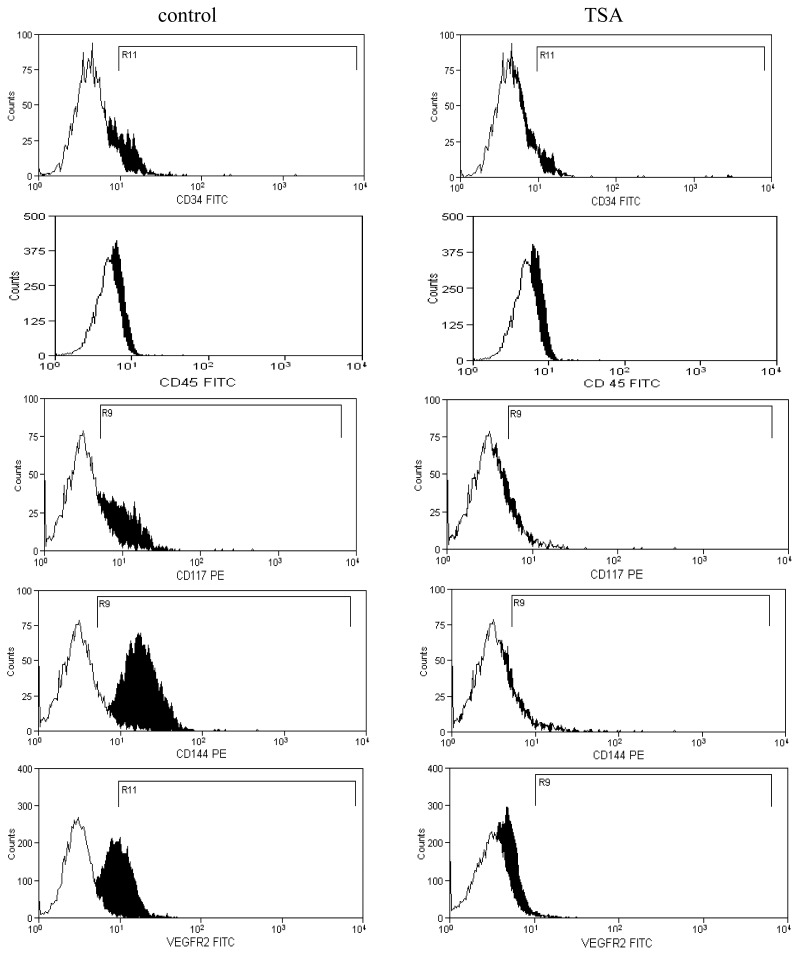
Flow cytometry analyses of EPCs treated with TSA (1 μM). Immunophenotype profile shows an inhibition of CD117 (94.3%), CD 144 (93.8%) and VEGFR2 (88.2%). Results are represented as mean ± standard error, *n* = 3, *p* < 0.05.

Our results show that TSA down-regulates the expression of *CD117*, *CD133*, *CD144*, *CXCR4*, *Tie-2*, and *VEGFR2*, whereas the expression of CD45 and CD34 remained unchanged, demonstrating that HDAC is involved in endothelial differentiation of progenitor cells (Figures 4 and 5). The expression of CD144 (VE-cadherin) was significantly decreased at mRNA level and also protein as shown by flow cytometry. A possible mechanism related to VE-cadherin expression may be explained by the impossibility of transcription factors such as HoxC6 to interact with acetylated histones and activating the VE-cadherin promoter [24]. *CXCR4*, *Tie-2*, and *VEGFR2* gene expression was significantly decreased by HDAC inhibitor TSA. A mechanism that could abrogate endothelial differentiation of progenitor cells is by reducing the expression of homeobox transcription factor (Hox) that are implicit in the activation of endothelial genes. Knockdown and overexpression studies revealed that *HoxA9* is a critical regulator of postnatal neovascularization and acts as a master switch to direct expression of the endothelial-committed genes [24–27].

In adult, fetal and progenitor stem cells recent studies show that HDAC inhibitors are also involved in stem cell pluripotency, but the mechanisms are still unclear [4,28]. It seems that HDAC inhibitors block the interaction of certain transcription factor with the genes involved in differentiation towards a specific cell line. In our study we want to see which genes are down-regulated in commitment of a particular cell type represented by endothelial progenitor cells derived from umbilical cord blood. Rössig *et al.*, shows that inhibitors of histone deacetylation downregulate the expression of endothelial nitric oxide synthase (eNOS) and compromise endothelial cell function and angiogenesis; also showing that HDAC inhibitors reduce the expression of *VEGFR2*, down-regulates *HoxA9* expression and EPC formation, and blocks the formation of a vascular network using *ex vivo* analysis of new vessel growth in the allantois assay [24]. Burba *et al.* showed that HDAC inhibitors determine changes in CD34^+^ phenotype due to activation of different pathways involved in cell proliferation and clonogenicity, and in modulation of stem cells markers such as CD34, CD38, CD133 and KDR [28]. Using small interfering RNAs, Mottet *et al.* observed that HDAC7 silencing in endothelial cells altered their morphology, their migration, and their capacity to form capillary tube-like structures *in vitro* but did not affect cell adhesion, proliferation, or apoptosis [29]. Let *et al.*, showed that global deacetylation of histones is necessary for *in vitro* differentiation of endothelial progenitor cells; removal of TSA from medium led to a 3.7-fold increase in the of alkaline phosphatase activity [30].

HDAC inhibitors are potent inducers of histone acetylation, a consequence of inhibition of HDAC activity and altering the cellular balance between HATs and HDACs, in favor of HATs [8]. Lagger *et al.* 2002, demonstrate that one of the key functions of HDAC1 is to prevent the expression of antiproliferative genes in cycling cells such as CDK inhibitors p21 and p27. Their findings indicate that deacetylases are essential for unrestricted cell proliferation by repressing the expression of selective cell cycle inhibitors [31]. Furthermore histone deacetylases inhibitors act as anti-angiogenic agents altering vascular endothelial growth factor (VEGF) signaling. TSA and SAHA were shown to prevent human umbilical cord endothelial cells (HUVEC) from invading a type I collagen gel and forming capillary-like structures, inhibiting the formation of a CD31-positive capillary-like network in embryoid bodies and angiogenesis in the CAM assay. TSA also prevented, in a dose-response relationship, the sprouting of capillaries from rat aortic rings. TSA inhibited in a dose-dependent and reversible fashion expression of VEGF receptors, VEGFR1, VEGFR2, and neuropilin-1 [32]. Thus HDAC inhibitors begin to be used as anti-cancer drugs in human cells. The Butyric Acid has been shown to induce classical maturation and anti-tumor effects in colon cancer cell lines, including the inhibition of cell proliferation and the stimulation of differentiation and apoptosis. In colon tumors expression of several HDACs are upregulated, by deregulation of fundamental signaling pathway β-catenin- TCF-*myc*[8].

Treatment with HDAC inhibitors have emerged as an important new class of potent anti-inflammatory agents in a number of cell types, including endothelial cells. For example, HDAC inhibitors have shown promise for the treatment of a growing number of chronic inflammatory diseases such as inflammatory bowel disease, systemic lupus erythematosus, and rheumatoid arthritis. So far the mechanism of action remains unclear but may involve modulation of NF-B transcriptional activity, in addition to chromatin-based mechanisms [33]. Interestingly a recent publication demonstrates that inhibition of HDACs increases dendritic sprouting, learning, and memory [34].

Elucidation of these mechanisms is expected to open new opportunities in the interface between chemistry and stem cell biology providing valuable tools to improve stem cell applications for tissue regeneration therapies.

## 3. Experimental Section

### 3.1. Cell Culture

Endothelial progenitor cells derived from human umbilical cord blood were obtained by Histopaque (Sigma-Aldrich, St. Louis, MO, USA) density gradient centrifugation at 400× *g*, for 30 min at room temperature as previously describe [11,13]. After centrifugation, the mononuclear cells (MNCs) layer was harvested and washed twice in Dulbeco’s Modified Eagle’s Medium (DMEM, Sigma-Aldrich) supplemented with 10% fetal bovine serum (FBS). The MNCs were plated on plastic dishes coated with fibronectin (1 μg/cm^3^, BD Biosciences, San Jose, CA, USA) in endothelial differentiation medium (MV2, Promocell, Heidelberg, Germany), supplemented with 15% FBS, 40 ng/mL vascular endothelial growth factor (VEGF), 100 μg/mL endothelial cell growth supplement, 100 U/mL penicillin, 100 μg/mL streptomycin, and 50 μg/mL neomycin (all purchased from Sigma-Aldrich). Cell cultures were maintained at 37 °C with 5% CO_2_ and 21% O_2_ in a humidified atmosphere. One day after plating, the non-adherent cells were discarded and fresh medium was applied. To maintain optimal culture conditions, medium was changed twice a week. For the proposed studies was used one characterized EPC line, at passages 6 and 7. For inhibition of HDACs we used 3 HDAC inhibitors: Butyrate (BuA, 2 mM), Trichostatin A (TSA, 1 μM), and Valproic acid (VPA, 0.5 mM) (Sigma-Aldrich, St. Louis, MO, USA).

### 3.2. Cell Proliferation and Cytotoxicity Assay

Cell proliferation and cytotoxicity of EPCs stimulated with HDACs inhibitors was asses using CellTiter 96^®^ Non-Radioactive Cell Proliferation Assay (Promega, Madison, WI, USA) according to the manufacturer’s specifications. We test different concentration of HDACs inhibitors (TSA 0.3 μM, 1 μM, 2.5 μM; VA 0.5 mM, 1 mM, 2mM; BuA 0.5 mM, 1 mM, 2 mM ) at different time interval. Briefly, 96 well plate with 2.500 EPCs were treated with different concentration of HDACs inhibitors for 24 h and 72 h. 15 μL of solution I was added in each well and incubate for 4 h, following by addition of solution II and incubate for another 1 h. The color intensity of formazan was determinate by spectrofotometry at 570 nm.

### 3.3. Reverse Transcription-Polymerase Chain Reaction (RT-PCR)

To assess the expression of endothelial genes involved in EPCs differentiation, cells grown in DMEM medium supplemented with 10% FBS were stimulated for 48 h with TSA (1 μM). Total RNA extraction was performed using GeneElute Mammalian Total RNA Miniprep Kit (Sigma-Aldrich) and reverse-transcription reaction was made using M-MLV polymerase (Invitrogen, Carlsbad, CA, USA); RT-PCR was assessed using a PCR kit (Promega, Madison, WI, USA), following manufacturer’s protocol. The sequences of *GAPDH*, *CXCR4*, *Tie-2*, *VE-cadherin*, and *CD133* primers (Metabion GmbH, Martinsried, Germany) are listed in Table 1. PCR reactions were carried out in a Corbett Thermal Cycler (Qiagen, Hilden, Germany) with the following schedule: denaturation step at 95 °C for 5 min, 35 cycles of amplification (denaturation at 94 °C for 45 s, annealing at 60 °C for 45 s, extension at 72 °C for 45 s), and incubation step at 72 °C for 10 min. Synthesized DNA fragments were detected by 1.5% agarose gel electrophoresis with ethidium bromide staining. Quantification was performed by densitometry using an Image Master Total Lab Software (Pharmacia Biotech, Buckinghamshire, UK). The experiments were done in triplicate, with controls represented by samples not stimulated with HDAC inhibitors.

### 3.4. Real Time-PCR Quantification of Telomerase Activity

Telomerase activity was determined using the telomerase amplification protocol assay performed with the TRAPeze@RT Telomerase Detection Kit (Chemicon, Temecula, CA, USA), according to the manufacturer’s instructions. Cells were grown to 70%–80% confluence and lyses with CHAPS buffer. For each reaction was used 1.5 μg protein. The real-time parameters includes 1 cycle at 30 °C, 30 min, 1 cycle at 95 °C 2 min and 45 cycles (94 °C 15 s, 59 °C 60 s, 45 °C 10 s).

### 3.5. Flow Cytometry

Expression of EPCs surface molecules was assessed by flow cytometry (MoFlo FACS, Dako, Glostrup, Denmark) using 1 × 10^5^ cells stained with fluorochrome-conjugated (Phycoerythrin, PE; Fluorescein isothiocyanate, FITC) antibodies against CD34, CD45, CD117, VEGFR_2_, VE-cadherin, and ICAM-1 (Dako). Accutase-detached cells were washed in phosphate buffered saline (1× PBS) and incubated for 30 min at 4 °C with either PE- or FITC-conjugated antibodies. For negative controls, the cells were stained with the corresponding isotype-matched IgGs (IgG1, IgG2a/b, Dako). Flow cytometry data were analyzed using the Summit 4.0 software (Dako).

### 3.6. In vitro Wound-Healing Assay

An equal number of cells (1 × 10^5^) were plated in triplicates on 6-well plates. Cells were grown in DMEM medium with 10% FBS, until the cells reached the confluence. The monolayer of confluent cultures was lightly scratched with a 1000 μL pipette tip and photographed by phase-contrast microscopy at timed intervals for up to 24 h. The assay was performed in triplicates with controls represented by EPCs grown in the same culture conditions, but without HDAC inhibitors. Quantification was done using the AxioVision software (4.8.1 version, Carl Zeiss MicroImaging GmbH, Jena, Germany) by measuring the number of pixels in the wound area and calculating the decrease in the scratched areas. This was achieved by subtracting the number of pixels at the 24 h time points from the number of pixels in the corresponding wound area at the 0 h time point.

### 3.7. Statistical Analysis

Statistical analysis was performed by a one-way analysis of variance (ANOVA) using the OriginPro 7.5 software. Differences were considered statistically significant when *p* < 0.05. The results are presented as means ± standard error (SE), where *n* represents the number of experiments.

## 4. Conclusions

The discovery of epigenetic differentiation programming gave rise to new treatment strategies, such as the mechanism of gene silencing being reversible. Certainly, pharmacological inhibitors of Class I and II HDAC activity have been identified as potent inducers of growth arrest, differentiation and apoptosis. HDAC inhibitors are able to potentiate both stem cell differentiation and somatic cell reprogramming to pluripotency. This may suggest that common mechanisms are involved in opposite changes of the differentiation status. Elucidation of these mechanisms is expected to open new opportunities in the interface between chemistry and stem cell biology.

The enzymatic activity of histone deacetylases is necessary for endothelial commitment of progenitor cells, HDAC inhibitors, leading to reduction of the proliferative and migratory activity of EPCs and reducing the expression of endothelial markers. HDAC inhibitors could prove to be valuable tools in improving stem cell applications for tissue regeneration therapies.

## Figures and Tables

**Table 1:** Sequences of the oligonucleotide primers (Metabion, Martinsried, DE) used for RT-PCR.

Gene	GeneBank accession number	Sequences of oligonucleotide primers	Predicted size (pb)
*GAPDH*	NM_002046	S: 5′-ACCACAGTCCATGCCATCAC-3′ A: 5′-TCCACCACCCTGTTGCTGTA-3′	450
*CXCR4*	NM_003467	S: 5′-GATGACAGATATATCTGTGACCGC-3′ A: 5′-TTAGCTGGAGTGAAAACTTGAAGA-3′	519
*Tie-2*	NM_000459	S: 5′-CATACTGGGGAAAGCAATGAAAC-3′ A: 5′-ACCACTGTTTTTCACCTTCCAAA-3′	281
*VE-cadherin*	NM_001795	A: 5′-CTTTGCCTCCAGGCAGATAG-3′ S: 5′-CCTTGGGATAGCAAACTCCA-3′	283
*CD133*	NM_006017	S: 5′-CAGTCTGACCAGCGTGAAAA-3′ A: 5′-GGCCATCCAAATCTGTCCTA-3′	200
